# NABhClassifier Server: A Tool for the Identification of Helical Nucleic Acid-Binding Sequences in Proteins

**DOI:** 10.1021/acs.jcim.4c02244

**Published:** 2025-02-22

**Authors:** Rogerio Margis, Iara Macedo, Nureyev F. Rodrigues, Mateus Dias-Oliveira, Fernanda Lazzarotto, Diego Trindade de Souza, Geancarlo Zanatta

**Affiliations:** † Postgraduate Programme in Cellular and Molecular Biology (PPGBCM), Center of Biotechnology, Federal University of Rio Grande do Sul, 90650-001 Porto Alegre, RS, Brazil; ‡ Postgraduate Programme in Genetics and Molecular Biology (PPGBM), Federal University of Rio Grande do Sul, 91501-970 Porto Alegre, RS, Brazil; § Department of Biophysics, Federal University of Rio Grande do Sul, 91501-970 Porto Alegre, RS, Brazil; ∥ Center of Biotechnology, Federal University of Rio Grande do Sul, 90650-001 Porto Alegre, RS, Brazil

## Abstract

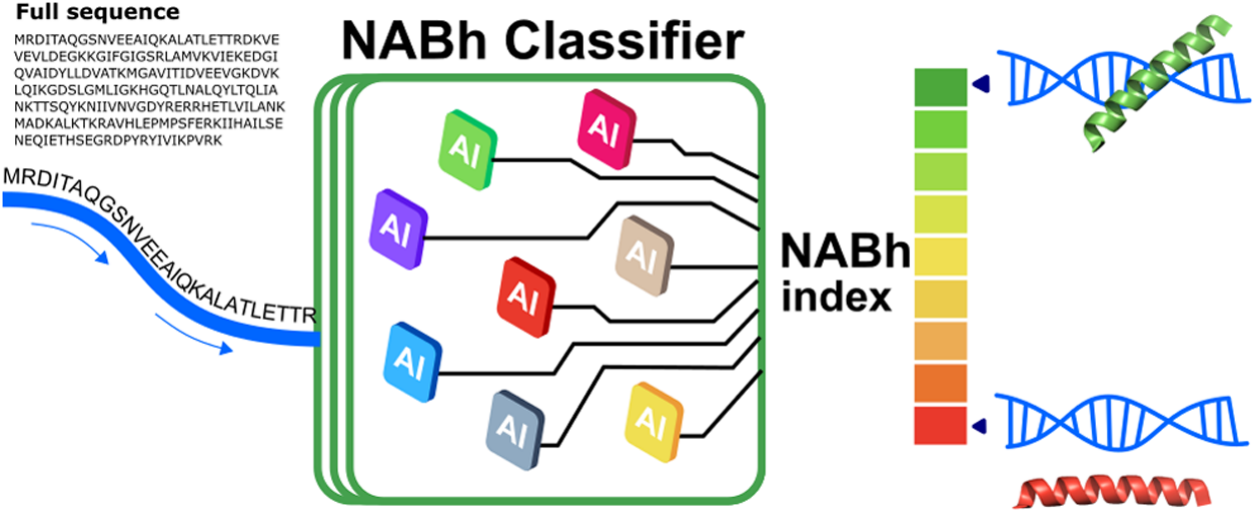

Engineered proteins capable of binding and transporting nucleic acids hold significant potential for advancing disease control in both the medical and agricultural fields. However, identifying small nucleic acid-binding domains remains challenging, as existing predictors primarily classify entire proteins as binders or nonbinders rather than targeting specific binding regions. Here, we introduce NABhClassifier, a highly efficient and precise web server designed to detect small helical sequences with nucleic acid-binding potential. Featuring an intuitive interface and a fully automated prediction pipeline, NABhClassifier integrates eight machine learning models for rapid analysis, delivering results in seconds per protein sequence. Predictions are summarized in the NABh index, a consensus score that combines outputs from all models for enhanced reliability. The server’s accuracy has been validated on data sets of DNA-binding and single- and double-stranded RNA-binding proteins from various species. NABhClassifier provides a powerful tool for identifying small helices with nucleic acid-binding capacity, facilitating the discovery of novel biotechnological applications. The server, along with tutorials, is freely accessible at http://143.54.25.149.

## 1 Introduction

The interest in the biotechnological application of nucleic acid-binding proteins (NABPs) has grown significantly in recent years. In nature, NABPs play essential roles in regulating key cellular processes. When engineered, these proteins have the potential to bind and transport small nucleic acid fragments for pharmacological and agricultural purposes.^1,2^ However, identifying the interaction regions responsible for the recognition process remains challenging and time-consuming.

Protein interactions with nucleic acids are crucial for maintaining and accessing genetic information, which is essential for numerous cellular processes including transcription, translation, and DNA repair. NABPs typically contain at least one DNA or RNA-binding domain, where interactions with amino acids occur in either specific or nonspecific manner.^3,4^ These proteins are generally classified into two categories based on their ability to bind DNA or RNA, although many NABPs can bind both exhibiting sequence and structural specificities. DNA-binding proteins are enriched with arginine, tryptophan, tyrosine, histidine, phenylalanine, and lysine residues, while they show depletion of glutamate, aspartate, and proline at the protein–DNA interface. RNA-binding proteins are enriched in arginine, methionine, histidine, and lysine residues, with depletion of glutamate and aspartate at the protein–RNA interface.^3,4^

Proteins that bind nucleic acids in a sequence-specific manner often contain well-defined structural motifs, such as zinc-fingers (which rely on cysteine and histidine residues for Zn^2+^ binding), leucine zippers (which require leucine residues), helix–loop–helix motifs (which contain conserved arginine, lysine, or histidine residues), and helix–turn–helix motifs (which have conserved “shs” and “phs” patterns, where ‘s’ represents a small residue, most commonly glycine in the first position, ‘h’ is a hydrophobic residue, and ‘p’ is a charged residue, typically glutamate).^3−5^

Conversely, many proteins recognize nucleic acids based on structural, rather than sequence specificity.^3^ Structure-specific proteins include G-quadruplex binding proteins (enriched in glycine, arginine, aspartic acid, asparagine, and valine, while depleted in cysteine, histidine, leucine, proline, glutamine, and tryptophan), cruciform binding proteins (enriched in lysine and serine, while depleted in alanine, glycine, glutamine, arginine, tyrosine, and tryptophan), triplex binding proteins (enriched in asparagine, aspartic acid, isoleucine, and tyrosine, while depleted in cysteine, histidine, and proline), and Z-DNA/RNA-binding proteins (enriched in isoleucine, aspartic acid, and lysine, while depleted in cysteine).^3,5^

NABPs can currently be identified and characterized through various experimental methods, such as pull-down assays, yeast one-hybrid systems, electrophoretic mobility shift assays, and chromatin immunoprecipitation.^6−9^ However, these techniques are often time-consuming and costly. With the increasing availability of protein sequence data and accumulated knowledge, computational approaches and prediction tools now allow for the identification of NABPs before their experimental validation.

One such protein with potential RNA-binding activity is KhpB, also known as EloR. It contains two RNA-binding domains: a KH domain (GXXG) and an R3H domain (RXXXH), as well as a Jag domain, which mediates protein–protein interactions.^1,10^ This combination suggests that KhpB functions as an RNA-binding protein, capable of directly binding to small RNAs.^1,2,11,12^ Given its ability to bind small RNAs, KhpB could potentially serve as a delivery protein for double-stranded RNA (dsRNA) molecules, which could be processed into small interfering RNAs (siRNAs). These siRNAs would then guide the RNA-induced silencing complex (RISC) to degrade target mRNAs (mRNAs), effectively silencing specific genes. This mechanism underlies RNA interference (RNAi), which is a powerful tool for gene modulation.

To advance research in this field, we developed a bioinformatics tool designed to identify nucleic acid-binding sequences in proteins. By pinpointing proteins with nucleic acid-binding capabilities, researchers can explore their potential applications, paving the way for innovative strategies in gene silencing and other RNA-related processes.

In this work, we introduce NABhClassifier, a web server designed to identify small nucleic acid-binding sequences within the helical domains of proteins. NABhClassifier automates the identification of helical secondary structures in protein sequences, streamlines the featurization process, and leverages artificial intelligence, integrating results from eight machine learning models for the identification and classification of nucleic acid-binding helical peptides.

## 2 Methods

### 2.1 Identifying and Extracting Helical Secondary Structures from Protein Sequences

The algorithm systematically scans the entire protein sequence for the presence of alanine, glutamate, histidine, lysine, leucine, methionine, glutamine, and arginine residues. When a six-residue segment contains at least four of these specified amino acids, it is classified as a potential helical structure. The minimum value of six was chosen because at least 6–7 amino acid residues are typically required to form a well-defined helical structure. Shorter segments (fewer than six residues) may still adopt a helical conformation but are often unstable. Subsequently, only helices consisting of six or more residues are extracted and advanced to the next step in the pipeline, where feature extraction is performed.

### 2.2 Featurization Process

For each input sequence, a total of 20 features are calculated. They are charged at pH 7.4, frequency of acid AA, frequency of all basic AA, frequency of all helix breaker, frequency of aromatic AA, frequency of basic AA, frequency of H-bond AA, frequency of hydroxyl-containing AA, frequency of nonpolar AA, frequency of ring containing AA, frequency of strong helix breaker, helix propensity, hydrophobicity index, hydropathy index, instability index, isoelectric point (pI), ligation to RNA, redox potential, sheet propensity AA, and turn propensity.

### 2.3 Training Data Sets

**Figure 1:**
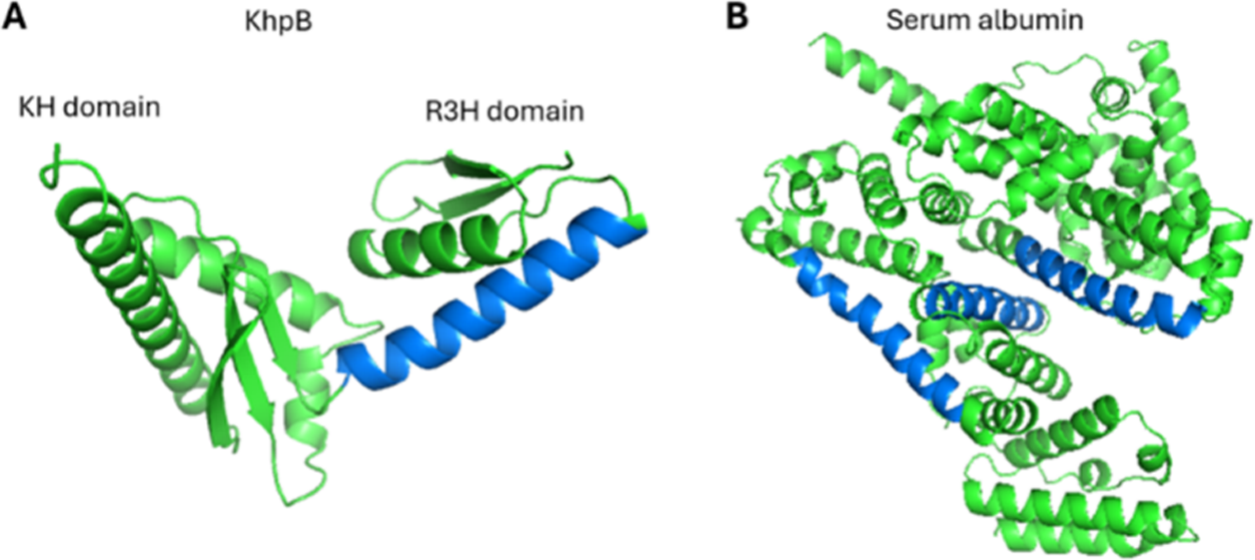
Cartoon representation of α helices that have been used during the training of multiple machine learning models. (A) Representation of the 22 amino acid region present in the R3H domain of the KhpB carboxy-terminal α helix (in blue) from 2500 different bacterial species used as TRUE sequences. (B) Representation of three α helices (in blue) extracted from serum albumin sequences and used as FALSE sequences.

For the classification task, models were trained using features calculated from 22 amino acid long helical sequences. The positive set was formed by segments extracted from the KhpB carboxy-terminal α helix from 2560 different bacterial species, while the negative set was formed by 701 α helices present in the albumin structure from 90 different organisms plus 1859 random generated sequences of 22 amino acids. The data set was divided into a training set (70%) for model learning and a test set (30%) to assess the generalization performance of the model. A graphical representation of KhpB and Albumin helices used for training is shown in Figure 1. Sequences of all proteins are provided in Supplementary File 1.

### 2.4 Machine Learning Models

The selected machine learning models encompass diverse classification approaches, ensuring robustness in capturing various data patterns. Linear models assess direct relationships between predictors and target variables, while SVMs handle high-dimensional data with optimized decision margins. Tree-based and ensemble models excel in detecting nonlinear patterns and managing imbalanced data sets, whereas nearest neighbor and probabilistic methods provide intuitive, distribution-based classifications. Neural networks and discriminant analysis enhance flexibility and class separability, while ensemble techniques integrate multiple classifiers to improve the overall performance. In total, 15 machine learning models were tested. They are Gradient Boosting Classifier (GBC), Support Vector Machine (SVM), Ada Boosting, Logistic Regression (LogReg), Ridge, Stacking, Random Forest (RF), Bagging, Multi-Layer Perceptron (MLP), Naive Bayes (NB), Bernoulli Naive Bayes (BNB), Linear Discriminant Analysis (LDA), Quadratic Discriminant Analysis (QDA), k-Nearest Neighbors (KNN), and Recurrent Neural Network (RNN). Hyperparameters were set using Grid search technique, and each model underwent cross-validation through 50 iterations. In Supplementary File 2, all possible combinations of hyperparameters for each ML model were tested and ranked to identify the best-performing configuration.

### 2.5 NABh Index as a Consensus Score

To account for variances among different machine learning models, an index representing the consensus score (*I*_NABh_) is calculated based on the frequency of positive results for a given sequence (eq 1):
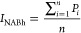
1where *I*_NABh_ is calculated by summing the number of positive predictions across the models for each instance and then dividing by the total number of models, *n* is the total number of models, and *P_i_* represents the TRUE prediction for each model *i*.

### 2.6 NABhClassifier Web Server Interface and Infrastructure

The NABhClassifier procedure is made available through an intuitive Web server interface (Figure 1). It allows users to easily upload and submit a sequence in a fasta format for analysis. The steps of sequence verification, helix extraction, and featurization are internaly automated and do not need user interference. A typical submission takes less than 2 s to be fully processed, and users can visualize sequences identified as TRUE NABhs through a report in the “Results” page.

NABhClassifier Web server is hosted in a 12-core virtual machine in the Data Processing Center (Centro de Processamentos de Dados, CPD) in the Federal University of Rio Grande do Sul. The code was written in python (ref), and the backend engine is implemented with Flask (https://flask.palletsprojects.com/en/stable/).

## 3 Results and Discussion

**Figure 2:**
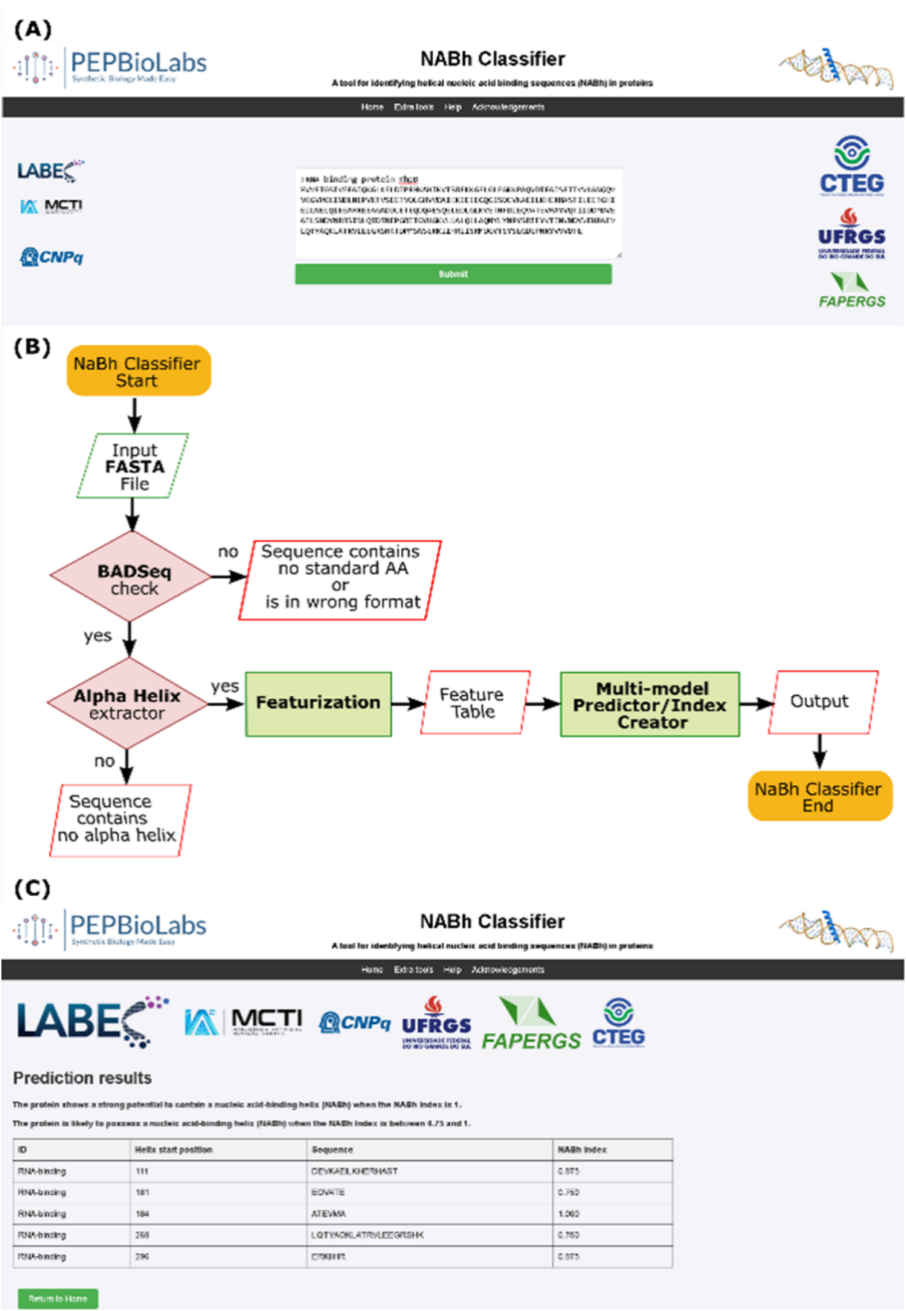
Overview of the NABhClassifier Webserver interface. (A) Home page of NABhClassifier allows users to input a sequence of interest and submit the job. (B) In the backend, the NABhClassifier Web server processes the analysis. (C) At the end, users can visualize results in the Results page and download resulting data in the csv file format.

The NABhClassifier Server is a powerful tool that accelerates the identification and development of small nucleic acid-binding peptides with helical secondary structures. This freely available web server is accessible to academic users and does not require specialized knowledge of protein biophysics or structural coordinates. Users simply input a FASTA sequence of a query protein (Figure 2A), after which the web server automatically preprocesses the sequence, identifies and extracts α helix segments, calculates molecular features, performs predictions, and classifies results according to the *I*_NABh_ index (Figure 2B). Helical sequences with an NABh index of 0.75 or higher are displayed on the “Results” page (Figure 2C).

**Figure 3:**
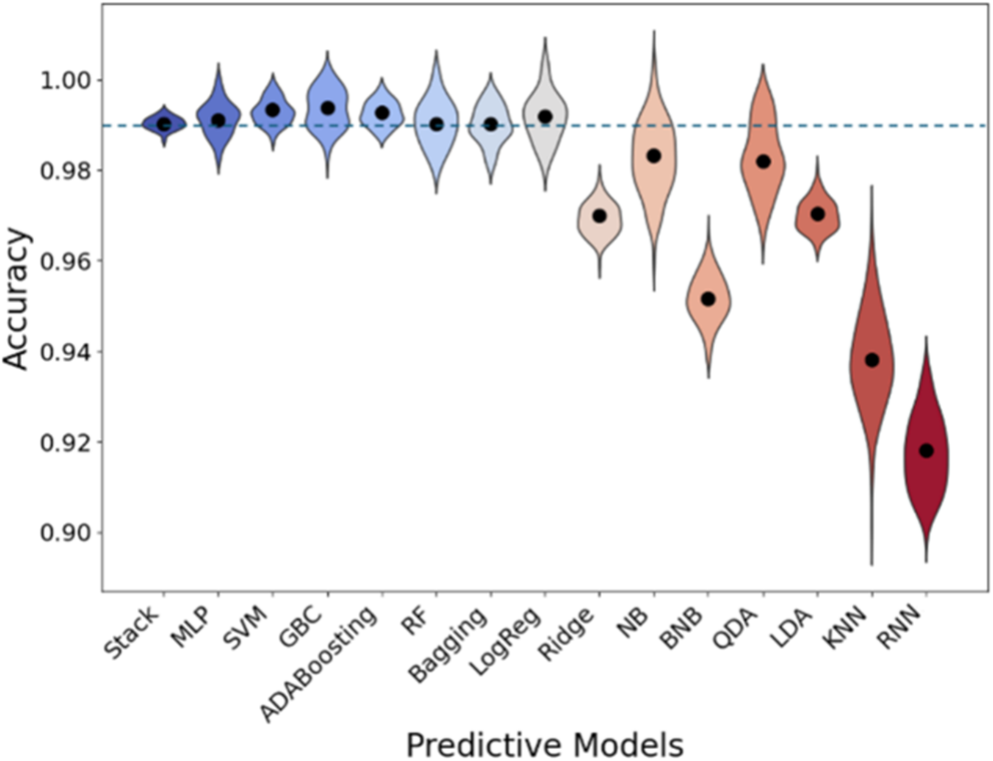
Graphical representation of the accuracy for the 15 models tested. Data are represented as violin plots, where the black dots represent the average accuracy and the outer shape of the violin represents the kernel density estimation (KDE) of the data, illustrating the probability density. Wider sections indicate more frequent values, while narrower sections indicate less frequent values. Note that the smooth shape of the violin is a result of the KDE process and may slightly extend beyond the data range (0–1) as a visual artifact.

Among the 15 ML models tested during training, eight demonstrated accuracy above 0.99 and were selected to form the NABhClassifier core (Figure 3). Combining these models outperformed individual models, improving the discrimination between true and false NABh sequences and enhancing the final prediction accuracy. Interestingly, adding more models beyond these eight did not further improve performance.

**Figure 4:**
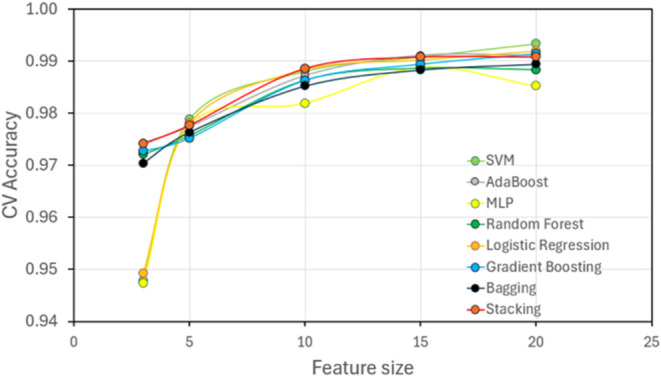
Demonstration of feature saturation across the top eight performing models.

To ensure consistency, each model was trained multiple times, with feature sets ranging from three to 20 molecular features. As shown in Figure 4, accuracy remained low with minimal features but reached a plateau around 99% when using 20 features. Table 1 provides a comprehensive overview of feature importance, highlighting that the “Frequency of basic AA”, “Redox potential”, and “Isoelectric point” collectively contribute 51.93% to the overall model relevance. Despite variation in individual feature weights across models, these three features play a dominant role in the classification performance, underscoring their critical importance.

**Table 1:** List of Features Average Relevance Using Eight Model Predictions

	feature	relevance (%)
1	frequency of basic AA	31.40
2	redox potential	12.73
3	isoelectric point (pI)	7.80
4	frequency of H-bond AA	4.74
5	instability index	4.62
6	charge at pH 7.4	4.46
7	frequency of all basic AA	4.25
8	ligation to RNA	4.16
9	frequency of nonpolar AA	3.93
10	turn propensity	3.30
11	helix propensity	3.12
12	frequency of ring containing AA	2.99
13	frequency of aromatic AA	2.10
14	hydropathy index	1.86
15	sheet propensity AA	1.81
16	hydrofobicity index	1.81
17	frequency of all helix breaker	1.80
18	frequency of strong helix breaker	1.11
19	frequency of acid AA	1.03
20	frequency of hydroxyl-containing AA	0.98

A detailed breakdown of the individual performance of each selected model is presented in Table 2. To enhance accuracy, we conducted 100 iterations of cross-validation, ensuring a robust estimation of each model’s performance metrics, including accuracy, precision, recall, *F*1 score, and AUC-ROC. While all models achieved high-performance metrics, approaching 0.99, analysis of confusion matrices revealed differences in how models classified true positives (TP), false positives (FP), false negatives (FN), and true negatives (TN), indicating model-dependent variations in sensitivity and specificity. Such discrepancies suggest that while the models generally perform well, their capacity to distinguish between true and false classifications varies, potentially impacting reliability across different application contexts. To address these inconsistencies, we developed a consensus metric, termed the NABh Index (*I*_NABh_, see eq 1). This index provides a confidence-based scoring system, where a score of 1.0 denotes unanimous hit identification by all eight models, 0.875 indicates agreement among seven models, and 0.75 indicates agreement among six models. Sequences scoring 1.0 exhibit a strong potential to behave as NABh, while sequences scoring between 0.75 and 1.0 indicate likely candidates. Predictions scoring below 0.75 are deemed to be unreliable for practical purposes.

**Table 2:** Model Performance Evaluation Matrices

model	accuracy	precision	recall	*F*1 score	AUC ROC	confusion matrix [[TP, FP], [FN, TN]]	cross-validation accuracy^a^	CVA std. dev.
Gradient Boosting	0.9915	0.9916	0.9915	0.9915	0.9993	[[748, 3], [10, 775]]	0.9933	0.0060
Support Vector Machine	0.9889	0.9891	0.9889	0.9889	0.9994	[[749, 2], [15, 770]]	0.9930	0.0055
Ada Boost	0.9948	0.9948	0.9948	0.9948	0.9995	[[748, 3], [5, 780]]	0.9925	0.0054
Logistic Regression	0.9922	0.9922	0.9922	0.9922	0.9995	[[746, 5], [7, 778]]	0.9919	0.0084
Stacking	0.9915	0.9916	0.9915	0.9915	0.9992	[[748, 3], [10, 775]]	0.9902	0.0072
Random Forest	0.9883	0.9884	0.9883	0.9883	0.9990	[[748, 3], [15, 770]]	0.9900	0.0076
Bagging	0.9870	0.9871	0.9870	0.9870	0.9994	[[747, 4], [16, 769]]	0.9891	0.0065
Multi-Layer Perceptron	0.9896	0.9896	0.9896	0.9896	0.9990	[[743, 8], [8, 777]]	0.9861	0.0102

a Cross-validation accuracy was calculated after 50 iterations.

### 3.1 Proof-of-Concept Validation

To validate the NABhClassifier server, we tested it on two case studies. In the first, NABhClassifier was challenged with a data set consisting of 458 single-stranded RNA-binding proteins (ssRBPs) and 474 double-stranded RNA-binding proteins (dsRBPs), initially identified by Sanchez de Groot et al.^13^ In the second case study (Table 4), NABhClassifier analyzed the coding sequences from the whole genome of five different organisms, *E. coli*-K12 (NCBI ASM584v2), *S. cerevisiae*-R64 (NCBI GCF_000146045.2), *C. elegans* (NCBI WBCel235), *A. thaliana* (TAIR10_pep_20101214), and *H. sapiens* (Ensembl Homo_sapiens.GRCh38.pep).

As shown in Table 3, NABhClassifier identified 23,607 helices across 474 dsRBP and 11,630 helices across 458 ssRBP, with at least one helix per protein analyzed. Among these, the NABhClassifier accurately identified approximately 75% (352) of dsRBP and 71% (327) of ssRBP proteins when using an *I*_NABh_ threshold of 1.0. When a threshold of *I*_NABh_ = 0.75 was applied, the classifier achieved a recovery rate of about 96% (454/474) for dsRBP and 95% (434/458) for ssRBP.

**Table 3:** Validation of True NABP Proteins Using the NABPh Classifier^a^

				TRUE index cutoff			TRUE recovery rate		
	proteins	with helix	predicted helix	1	0.875	0.750	1	0.875	0.750
dsRBP	474	474	23607	352	417	454	0.743	0.880	0.958
ssRBP	458	458	11630	327	408	434	0.714	0.891	0.948

a List of protein and transcript pairs analyzed with RPISeq and catRAPID in Supplementary Data 4 from Sanchez de Groot et al.^13^

In the second case study (Table 4), a whole-genome data set including five different organisms was employed. NABPs were identified using functional keywords such as “polymerase”, “RNase”, “helicase”, “nuclease”, “DCL”, “argonaute”, “RNA-binding”, “DNA-binding”, “histone”, “RNPs”, “chromatin”, “snRNP”, “ribosomal protein”, “Transcription factor”, and “Transcription_factor”. NABhClassifier recovered at least one helix per NABP and correctly identified 93–99% true NABPs employing an *I*_NABh_ = 0.75; 83–97% at *I*_NABh_ = 0.875; and 58–79% at *I*_NABh_ = 1. A detailed view of these data sets is shown in Supplementary File 3, which shows only classes of truly NABps among the analyzed organisms. NABhClassifier obtained a full recovery rate for DCL and AGO, both derived from *C. elegans*, when employing *I*_NABh_ = 1. Six complete recoveries were observed at *I*_NABh_ = 0.875, while four were identified at *I*_NABh_ = 0.75. However, in two classes—Polymerase and RNA-binding—proteins were not fully recovered at the tested threshold.

**Table 4:** NABP Identification Effectiveness in Different Organisms^a^

					TRUE index cutoff			TRUE recovery rate		
organism	all prot	NABP	% NABP	helix-containing protein	1	0.875	0.75	1	0.875	0.75
*E. coli*-K12	4298	398	9.26	398	228	332	370	0.573	0.834	0.930
*S. cerevisiae*-R64	6014	335	5.57	335	266	325	331	0.794	0.970	0.988
*C. elegans*	28587	685	2.40	685	532	619	656	0.777	0.904	0.958
*A. thaliana*	48320	3326	6.88	3326	2418	3002	3206	0.727	0.903	0.964
*H. sapiens*	136194	6379	4.68	6379	4878	5804	6142	0.765	0.910	0.963

a NABP were identified using keywords search in the proteome annotation of each species.

Despite growing interest in NABPs for biotechnological applications, identifying viable small domains with the ability to bind and transport nucleic acids remains challenging. NABPs generally contain at least one DNA or RNA-binding domain, enabling specific or nonspecific interactions with amino acids. They are often categorized by their binding ability to DNA or RNA, although many NABPs can bind both and exhibit specificity based on sequence or structure. Typically, DNA-binding proteins are enriched with residues arginine, tryptophan, and lysine, while RNA-binding proteins are enriched with arginine, methionine, and histidine.^3^

Experimental NABPs characterization methods, including pull-down assays, yeast one-hybrid systems, electrophoretic mobility shift assays, and chromatin immunoprecipitation, are time-intensive and costly. The growing availability of protein sequence data and structural information has facilitated the development of computational algorithms designed to identify nucleic acid-binding sites in proteins and to model protein–NA interactions. Among sequence-based prediction tools, catRAPID omics v2 leverages the use of organism-specific proteomic and transcriptomic libraries to allow the high-throughput prediction of protein–RNA interactions,^14^ while GraphSite employs a transformer model that combines AlphaFold-predicted structures and sequence data, focusing on DNA-binding residue prediction, but also with some RNA recognition capabilities.^15^ In this concern, while other sequence-based and structure-aware algorithms focus on the identification of nucleic acid-binding sites on proteins, the NABhClassifier server also identifies NABPs based only in the sequence, in addition to extracting small helical peptides with the ability to bind nucleic acids. The server offers an easy-to-use input interface and takes less than 2 s to analyze a single fasta sequence and delivers the results, offering a fast and highly accurate prediction. Although NABhCassifier was trained solely on KhpB carboxy-terminal helices from across 2560 different bacterial species, it was able to correctly identify NABPs across different protein classes, from *E. coli*, *S. cerevisiae*, *C. elegans*, *A. thaliana*, and *H. sapiens*. With an intuitive interface and fully automated prediction pipeline, NABhClassifier leverages eight machine learning models for rapid analysis, generating results within seconds per protein sequence. Predictions are summarized in the NABh index, a consensus score that combines outcomes from all models for enhanced reliability. The server’s accuracy has been validated on DNA-binding and single- and double-stranded RNA-binding protein data sets from different species. NABhClassifier provides a robust framework for detecting small helices with nucleic acid-binding capacity, advancing discoveries in biotechnology.

## 4 Conclusions

In this study, we present NABhClassifier, a novel computational tool designed to identify nucleic acid-binding proteins (NABPs) and extract small helical peptides capable of binding to single- and double-stranded RNA. Our approach integrates eight machine learning models and introduces the *I*_NABh_ index, which accounts for variations among models and provides accuracy assessments at three confidence levels. Compared to existing sequence-based methods, the NABhClassifier offers superior performance, delivering high-accuracy predictions in under two seconds. Additionally, by identifying small helical peptides within protein structures, the tool surpasses the limitations of conventional algorithms, which focus solely on binding cavities. This approach enhances predictive accuracy and provides valuable biological insights into protein functionality.

## Data Availability

NABhClassifier server is freely available at http://143.54.25.149.

## Supporting Information Available

List with the KhpB carboxy-terminal α helix sequences used as TRUE set and a list of albumin and random helical sequences used as FALSE set during training (Supplementary File 1) (XLSX)
Best combination of hyperparameters identified during the Grid Search Hyperparameters Adjustment procedure (Supplementary File 2) (XLSX)
Performance of NABhClassifier across various protein classes in different organisms (Supplementary File 3) (Table S1) (PDF)
