# Supplementary material for: NABhClassifier Server: A Tool for the Identification of Helical Nucleic Acid-Binding Sequences in Proteins
Source: J Chem Inf Model. 2025 Feb 22;65(5):2361–7. doi: 10.1021/acs.jcim.4c02244 (PMC11898072; doi:10.1021/acs.jcim.4c02244)
Supplement: Supplementary file 3 — ci4c02244_si_003.pdf [file ci4c02244_si_003.pdf]

NABhClassifier Server: a tool for the identification of helical nucleic acid binding sequences in proteins

Rogerio Margis [a,b,c], Iara Macedo [b], Nureyev F. Rodrigues [c], Mateus Dias-Oliveira [a], Fernanda Lazzarotto [d], Diego Trindade de Souza [c], Geancarlo Zanatta [ac]*

a - Postgraduate Programme in Cellular and Molecular Biology (PPGBCM), Center of Biotechnology, Federal University of Rio Grande do Sul, 90650-001, Porto Alegre, RS, Brazil.

b – Postgraduate Programme in Genetics and Molecular Biology (PPGBM), Federal University of Rio Grande do Sul, 91501-970, Porto Alegre, RS, Brazil.

c - Department of Biophysics, Federal University of Rio Grande do Sul, 91501-970, Porto Alegre, RS, Brazil.

d – Center of Biotechnology, Federal University of Rio Grande do Sul, 90650-001, Porto Alegre, RS, Brazil.

*** Corresponding Author**

Supplementary Table 1 - NABP identification effectiveness across various protein classes in different organisms.

	E.coli				*S.cerevisiae*				*C.elegas*				*A.thaliana*				*H.sapiens*			
Protein classes	Count	1	0.825	0.75	Count	1	0.825	0.75	Count	1	0.825	0.75	Count	1	0.825	0.75	Count	1	0.825	0.75
Polymerase	34	25	30	32	54	36	49	51	143	114	127	136	375	301	346	355	752	527	641	716
Helicase	18	17	17	**18**	72	69	**72**	72	130	122	128	**130**	263	235	256	262	823	772	816	818
Nuclease	59	34	48	53	48	37	47	**48**	138	109	129	131	435	294	375	410	846	439	720	789
DCL – Dicer-like	ni	ni	ni	ni	ni	ni	ni	ni	1	**1**	1	1	20	15	**20**	20	23	20	**23**	23
AGO - Argonaute	ni	ni	ni	ni	ni	ni	ni	ni	9	**9**	9	9	10	**10**	10	10	26	24	**26**	26
RNA-binding	5	3	3	3	51	41	49	50	45	27	39	40	521	327	454	487	768	604	731	759
DNA-binding	267	139	221	251	30	25	29	**30**	22	16	16	20	804	551	703	785	461	303	378	407
Histone	ni	ni	ni	ni	44	30	**44**	44	184	127	159	177	212	145	194	210	1022	905	998	1008
Transcription factor	ni	ni	ni	ni	46	37	**46**	46	ni	ni	ni	ni	691	548	646	672	1846	1461	1661	1786
Total protein	383	218	319	357	345	275	336	341	672	525	608	644	3331	2426	3004	3211	6567	5055	5994	6332
Percent. (%)		57	83	93		80	97	99		78	91	96		73	90	96		77	91	96
